# Effectiveness of micronutrient-fortified rice consumption on anaemia and zinc status among vulnerable women in Bangladesh

**DOI:** 10.1371/journal.pone.0210501

**Published:** 2019-01-10

**Authors:** Gulshan Ara, Mansura Khanam, Ahmed Shafiqur Rahman, Zhahirul Islam, Shahriar Farhad, Kazi Istiaque Sanin, Sihan Sadat Khan, Mohammad Mahbobor Rahman, Herma Majoor, Tahmeed Ahmed

**Affiliations:** 1 icddr,b, Dhaka, Bangladesh; 2 United Nations World Food Programme, IDB Bhaban, Begum Rokeya Sarani, Dhaka, Bangladesh; University of Dhaka, BANGLADESH

## Abstract

Micronutrient deficiency is one of the biggest public health concerns in Bangladesh. As per World Health Organisation (WHO) in the 2016 report, 40% women of reproductive age suffer from anaemia. According to the National Micronutrient Survey 2011–2012, 57% women suffer from zinc deficiency. The objective of the present study was to determine the effectiveness of fortified rice (FFR in addressing anaemia and zinc deficiency among vulnerable women. Baseline and endline surveys were conducted among female Vulnerable Group Development (VDG) beneficiaries in five districts in Bangladesh before and after 12 months of FFR distribution. The intervention group received 30 kg FFR; the control group received 30 kg non-FFR for every month from January 2013 to December 2013. The sample sizes were 870 women (435/group) at baseline and 800 (400/group) at endline. Difference-in-difference (DID) was estimated to measure the effect of FFR on anaemia and serum zinc. In the baseline survey, 39% of the FFR group and 34% of the non-FFR group had anaemia. At endline, 34% of women in the FFR group were anaemic compared to 40.7% in the non-FFR group. At endline, prevalence of anaemia was reduced in the FFR group by 4.8% but increased in the non-FFR group by 6.7%. The DID estimation showed the reduction in anaemia after 12 months of FFR consumption was significant (*p* = 0.035). The DID in mean haemoglobin level after 12 months of FFR consumption was also statistically significant (*p* = 0.002). Zinc deficiency decreased by 6% in the FFR group at endline, though the DID was not significant. Most of the respondents of the FFR group reported that they received their entitled rice on a regular basis however only half of the non-FFR respondents received every month in 12 months. Anaemia was significantly associated with not consuming fortified rice, geographical region, older age and heavy menstrual bleeding (P<0.05). FFR reduced anaemia and zinc deficiency prevalence. Replacement of regular rice with FFR in the VGD programme is recommended to reduce anaemia among vulnerable groups.

## Background

Micronutrient deficiency, also known as hidden hunger, is currently a significant global health burden. Unlike protein energy malnutrition (PEM), the impacts of micronutrient deficiency are not always acutely visible and therefore termed as ‘hidden hunger’ [1,2]. Micronutrient deficiency negatively affects key developmental outcomes, including physical and mental development [3]. In extreme cases it can lead to vulnerability to disease, mental retardation, blindness and general loss in life-long productivity [3].

Micronutrient deficiencies are prevalent in Bangladesh across all wealth quintiles. However, pregnant women and children are most vulnerable. Anaemia is one of the biggest public health concerns in the country, where over 50% of young children (under 5-years-old) [4], 11% of school-aged children [5] and over 50% the women of reproductive age suffer from anaemia [6]. This high burden of deficiency has a far-reaching economic impact; 8% of the gross domestic product of Bangladesh is estimated to be lost due to anaemia alone [7]. Moreover, the 2011–12 National Micronutrient Survey found that the national prevalence of zinc deficiency was approximately 45% among preschool-age children. The deficiency was even higher among slum dwelling preschool children (52%) and among non-pregnant non-lactating women (66%) [8]. Many factors have been reported to contribute to micronutrient deficiencies, including diets with low nutrient quality and diversity, low household purchasing power, inadequate access to drinking water and sanitation facilities, inadequate knowledge of nutritional practices and inequality [8].

Fortification of staple foods is a proven and effective way [9] to ensure that many consumers, including women and children at risk of vitamin and mineral deficiencies, receive the micronutrients they require. Globally, the use of fortified rice has improved micronutrient intake and reduced micronutrient deficiencies in several countries. A six-month study to test the efficacy of iron-enriched rice with extruded iron premix rice (IPR) using ferrous sulphate as fortification among the anaemic school children aged 6–9 years in the Philippines, found that consumption of iron enriched rice positively reduced anaemia by 38% from baseline to endline [10]. Similar results were reported by the efficacy studies carried out in India, Egypt, Brazil and Mexico among infants, children and working women [11–13] providing global evidence that the consumption of fortified rice reduces micronutrient deficiencies.

Although a wider variation in dietary intake exists in Bangladesh, depending on socioeconomic status and urban/rural residence; the habitual diet is dominated by cereals which contribute to three-fourth of total calorie intake. Furthermore, the consumption of non-rice nutrient-rich food items, such as milk, meat, fish, fruits and vegetables, are highly price sensitive compared to consumption of rice. Milled rice is a poor source of micronutrients [2], yet milled rice consumption in Bangladesh is as high as 438 g per person per day, representing 77% of daily caloric intake. This is because rice is both affordable and its consumption is culturally engrained. While there are ongoing efforts to promote a diversified diet, rice will continue to remain a major component of the diet for Bangladeshi people. Therefore, consumption of fortified rice might be a cost-effective approach for providing the micronutrient requirements to a large segment of the population, especially the poorest and most vulnerable groups.

However, fortified rice cannot solve the issue of micronutrient deficiency alone, it represents a key part of the solution to ameliorate the burden of undernutrition [14]. The World Food Programme pioneered the introduction of micronutrient-fortified food in Bangladesh such as micronutrient-fortified rice (FFR), flour and biscuits to improve micronutrient status of vulnerable women, pre-primary and primary school children [15]. The Vulnerable Group Development (VGD) programme is the largest social safety net of the Government of Bangladesh that exclusively targets ultra-poor women and their households. The selection criteria for ultra-poor women includes widowed, separated/deserted/divorced or has a husband who is unable to work; has severe food insecurity; landless or owns less than 0.5 acre of land; has very low and irregular family income or works as casual labour; from a household headed by a woman [16]. The overall objective of the VGD Programme is to contribute to national initiatives towards ending hunger, achieving food security, improving nutrition and promoting sustainable development goals. Under the VGD programme, a monthly ration of 31.25 kg wheat or 30 kg rice over two years were distributed to rural ultra-poor women to ensure food security in their households [15]. WFP provides technical assistance to the Government of Bangladesh to strengthen the VGD programme. The project distributed FFR to 500,000 extremely vulnerable women and children from 2014 to 2016. The micronutrient mix used for fortifying the FFR includes vitamin A, vitamin B1, vitamin B12, folic acid, iron and zinc. The present study aimed to examine the effectiveness of rice fortification on anaemia and zinc deficiency among vulnerable women in Bangladesh.

## Methods

### Study design and participants

A community based longitudinal (controlled before-after) effectiveness study was conducted in 10 upzilas/subdistricts [17] of 5 districts of Bangladesh to evaluate the intervention provided by the WFP on the VGD beneficiaries and compared the outcomes at two time points; baseline and endline periods. The sampling was done among the VGD beneficiaries who received either fortified rice in 5 upazilas namely Kaligonj, Sarankhola, Tungipara, Dacope and Shyamnagar in the FFR group and unfortified rice rations in the non-FFR group. The 5 FFR upazilas were selected by the World Food Programme from 5 districts in different geographic locations across the country. The non-FFR upazilas were selected from the same districts with similar socio-economic background. A systematic random sampling method was employed to enrol the required number of participants for the baseline and endline surveys from the total list of VGD women in both the FFR and non-FFR upazilas. Participants for FFR group were drawn from the total list of approximately 15,000 VGD beneficiaries from 40 unions under the 5 upazilas. Similarly, participants for non-FFR group were selected from enlisted approximately 15,000 VGD women from 53 unions of the 5 upazilas. During the endline evaluation, similar sampling approach was employed, and participants were allocated to FFR and non-FFR group from the same sampling frame. However, the participants of baseline and endline survey were different. Baseline data collection was commenced from December 2014 to April 2015. After the baseline data collection, the rice distribution was not immediately initiated. Due to delay in fortified rice production, the onset of the intervention was also deferred for around 12 months. After 12 months of FFR/non-FFR consumption, the endline data was collected from December 2016 to April 2017.

### The micronutrient composition of fortified rice

The production of fortified rice in this project took place in two steps- i)the production of fortified rice kernels, which were made from cheaper rice flour mixed with micronutrients, reconstituted via hot extrusion technology, and ii) the homogeneous blending of fortified rice with un-fortified rice, usually at a 1:100 ratio. The estimated cost implication is an additional 3–5 percent at the retail level when compared with un-fortified rice [2]. The micronutrient fortificants used to fortify the rice includes Vitamin A, Vitamin B_1_, Vitamin B_12_, folic acid, iron, and zinc as shown in Table 1 [18].

**Table 1:** The micronutrient content per 100 g fortified rice.

Micronutrient	Amount
Vitamin A	150 μg
Iron	6 mg
Vitamin B_1_	0.4 mg
Folic acid	130 μg
Zinc	4 mg
Vitamin B_12_	1μg

### Sample size calculation

The sample size was calculated to detect a minimum statistically significant difference (10%) in the prevalence of anaemia between the intervention and control groups in the endline survey after one year of intervention (Table 2) using the following formula:

$$n=\frac{{\left[Z_{\alpha }\sqrt{2PQ}-Z_{1-\beta }\sqrt{P_{1}Q_{1}+P_{2}Q_{2}}\right]}^{2}}{{\left(P_{2}-P_{1}\right)}^{2}}\times Factor\;to\;adjust\;for\;drop\;out$$

[19]

Where *n* = required sample size for each survey, expressed as number of units of analysis;

*P*_1_ = proportion in the pre—intervention (or baseline) survey;

*P*_2_ = proportion in endline survey;

(*P*_2_ − *P*_1_) = expected difference between baseline and endline surveys, using the following parameters:

P=(P1+P2)2 and *Q* = (1 − *P*), *Z*_*α*_ = 1.96 at *α* = 0 − 0167 and *Z*_1−*β*_ = (−0.842) for the power of the test set at 0.85 and factored to adjust for drop out of 15%. The prevalence for anaemia was acquired from the Bangladesh Demographic Health Survey, 2011.

**Table 2:** Survey sample size calculations.

Target group	Indicator	Baseline survey (%)	Endline survey (%)	Minimum number samples in intervention group	Minimum number of samples in control group
**Women (15–49 years)**	Anaemia	42	31	400	400

Source: Bangladesh and Demographic Health Survey 2011

### Inclusion/Exclusion criteria

Predefined inclusion and exclusion criteria were employed during enrolment of the VGD beneficiaries. The inclusion criteria includes (i) women aged 15–49 years-old, (ii) possession of VGD programme card and (iii) provision of written consent agreement with their household head to participate in the study. The exclusion criteria were (i) known or suspected chronic or congenital disease, (ii) pregnancy and (iii) reported severe anaemia. The severe cases were suggested to visit the government health facility.

### Socioeconomic status

Socioeconomic status [20] was calculated as previously described using the Demographic Health Statistic Wealth Index to divide surveyed households into five socioeconomic quintiles, with quintile 1 being the lowest and quintile 5 being the highest. A list of variables, including homestead; land under cultivation; construction materials of the walls, roof and floor of the house; ownership of household assets (electricity, radio, television, mobile phone, land phone, chair, watch, table, cupboard, rickshaw, van, animal drawn cart, refrigerator, motor boat) and type of toilet facility, was considered.

### Morbidity

Each participating woman was asked about recent illnesses in the previous two weeks. Diarrhoea was defined as three or more abnormally loose or liquid stools without blood in the last 24 hours or any number of stools with blood (dysentery). Questions relating to menstrual problems, including the absence of periods, painful periods, heavy periods and irregular periods, were also included.

### Food frequency for last seven days

Respondents were asked about the frequency of consumption of different food items over a recall period for the last seven days. Food items were grouped into eight standard food groups over a maximum of seven days per week. Food groups included staples, pulse, vegetable, fruit, animal, sugar, dairy and oil.

### Operational Efficiency and compliance measure

An operational efficiency and compliance measuring tools were used at endline. The tools include a series of structured questions in different sections. Respondents were asked about type of rice, frequency of rice distribution, mode of packaging, timeliness in distribution, quantity, quality, loss and/or leakage of rice commodity. To measure compliance, women were asked about utilization of rice, consumption pattern, amount of rice purchased in last two months prior to starting of endline data collection, number of times they received rice in last 12 months etc. As this was a Government operated programme, the World Food Programme and relevant government authority followed the compliance using a structured monitoring tool icddrb collected compliance data during endline evaluation. International Centre for Diarrhoeal Disease Research, Bangladesh (icddr,b) was only responsible for baseline and endline data collection.

### Collection, preparation, transport and storage of biological samples

Trained phlebotomists collected peripheral blood samples from survey respondents during both the baseline and endline evaluations. After taking consent and maintaining aseptic precautions, about 5 mL of venous blood was collected and aliquoted into appropriate tubes with or without anticoagulant. Samples were transported to the laboratory at icddr,b in Dhaka, Bangladesh, twice a week to assess concentrations of zinc, C-reactive protein (CRP) and haemoglobin. As most of the study sites were hard to reach, it was not possible to transfer the samples immediately to the icddr,b laboratory. The samples were first preserved in -20°C then transferred to icddr,b laboratory maintaining to the cold. All samples were transported to a nearby temporary laboratory set up at a clinical setting for temporary storage, before being transferred to icddr,b. Haemoglobin concentration in whole blood was measured using the cyanmethaemoglobin method [21]. Serum samples were stored at -20°C until serum zinc was estimated by atomic absorption spectrophotometry [22] and serum CRP was determined via an immunoturbidometric method using a Roche automated clinical chemistry analyser Hitachi 902 [23].

### Definition of outcome variables

Anaemia is defined as haemoglobin level <12.0gm/dl in non-pregnant and non-lactating (NPNL) women [24]. Zinc deficiency is defined as serum zinc level of <10.10 mmol/L in NPNL women [25]. The adjustment for elevated CRP (>10.0 mg/l) was done by a mathematical correction [26].

### Statistical analysis

Stata software (version 13; Stata Corporation, College Station, TX, USA) was used for all univariate and bivariate analysis. Frequencies and percentages (for categorical variables) or means and standard deviations (for continuous variables) were calculated for descriptive statistics. The Student’s *t*-test and the chi-square test were applied to compare means and to explore the associations between categorical variables respectively. *P*-value <0.05 was considered significant for all tests. The difference-in-difference (DID) analysis was performed to measure the effect of the fortified rice distribution programme on anaemia and serum zinc concentration. Multivariable logistic regression was done by using the stepwise backward method to determine the factors significantly associated with anaemia and zinc deficiency.

### Ethical approval

This study (PR-14104) was approved by the Research Review Committee and Ethical Review Committee, the two compulsory components of the institutional review board of the icddr,b. Written informed consent was obtained from all study participants (>18 years) and/or assent (<18 years) from their parents/guardians prior to enrolment. The respondents were informed that their participation was voluntary, and they could withdraw themselves at any point during the interview.

## Results

### Socio-demographic characteristics of the households of VGD women

In Table 3, the age distributions of the women in the FFR (intervention) and the non-FFR (control) groups were statistically not significantly different; median age was 32 years at baseline (*p* = 0.490) and 34 year at endline (*p* = 0.531). More than one-fourth of the women in both groups had no education. About 60% of the households in both groups had access to electricity. More than 90% of the households, either the respondents or members of their household, had a mobile phone. Half of the households had one room for sleeping and the average number of people sleeping in a room was 1.6 at baseline and 1.5 at endline. More than 80% of the households in both groups were landless. Almost all the households were headed by women. Around 30% of the households in both groups obtained drinking water from a tube well. One third of beneficiary households in the non-FFR area and 16% in the FFR area drank water from ponds. Nearly one-third of the households in both groups had access to water-sealed sanitary latrines with a flush. However, more than 60% of the households in both groups used sanitary latrines without any flush system.

**Table 3:** Socio-demographic characteristics of the FFR and non-FFR groups.

	Baseline			Endline		
Characteristic	FFR	Non-FFR	*P-*value	FFR	Non-FFR	*P-*value
	*n* = 435	*n* = 435		*n* = 397	*n* = 409	
Age of respondents mean	32.45	32.44	0.49	34.13	34.7	0.53
Family size mean	4.5	4.6	0.12	4.6	4.5	0.12
Education of respondents, *n* (%)						
No education	119 (27.4)	109 (25.1)	0.05	109 (27.5)	122 (29.8)	0.04
Primary incomplete	112 (25.7)	132 (30.3)		74 (18.6)	97 (23.7)	
Primary complete	81 (18.6)	79 (18.2)		78 (19.7)	80 (19.6)	
Secondary incomplete	107 (24.6)	108 (24.8)		127 (32.0)	96 (23.8)	
Secondary complete/above	16 (3.7)	7 (1.60)		9 (2.3)	14 (3.4)	
Household assets, *n* (%)						
Electricity	259 (59.5)	244 (56.1)	0.17	274 (69.0)	268 (65.5)	0.33
Mobile phone	373 (85.7)	359 (82.5)	0.12	366 (92.1)	382 (93.4)	0.22
Number of rooms used for sleeping, *n* (%)						
1	213 (49.0)	234(53.8)	0.39	224 (56.4)	233 (57.0)	0.07
2	160 (36.8)	147(33.8)		139 (35.0)	132 (32.3)	
>3	62 (14.2)	54 (12.4)		34 (7.1)	45 (11.0)	
Number of sleeping rooms (Mean ± SD)	1.61±0.80	1.69±0.83	0.16	1.54 ±0.73	1.57±0.81	0.58
Type of homestead, *n* (%)						
Own homestead	357 (82.1)	345(79.3)	0.33	366 (92.2)	364 (89.0)	0.62
Land ownership, *n* (%)						
Homestead land	94 (21.6)	66 (15.2)	0.01	60 (15.1)	38 (9.3)	0.03
Agricultural land, *n* (%)						
Landless	372 (85.5)	351(80.7)	0.34	337 (84.9)	371 (90.7)	0.00
1–15 Decimal^a^ [27–29]	37 (8.50)	46(10.5)		24 (6.04)	16(3.91)	
>15	26 (5.9)	38 (8.7)		36 (9.1)	22 (5.4)	
Occupation of the respondents, *n* (%)						
Professional	20 (4.6)	17(3.9)	0.00	27(6.8)	24 (5.9)	0.00
Unskilled worker	49 (11.26)	34 (7.82)		18 (4.5)	21 (5.1)	
Agricultural day labourer	1 (0.23)	6 (1.38)		5 (1.3)	6 (1.5)	
Home servant	25 (5.52)	6 (1.38)		14 (3.5)	21 (5.1)	
Housewife	314 (72.18)	357 (82.07)		327 (82.3)	323 (79.0)	
Others	26 (5.97)	15 (3.44)		6 (1.5)	14 (3.4)	
Gender of household head, n (%)						0.11
Male	19 (4.37)	8 (1.84)	0.03	4 (1.01)	11 (2.69)	
Female	416 (95.6)	427 (98.16)		393 (98.99)	398 (97.31)	
Source of drinking water, n (%)						
Own tube well	317 (72.7)	230 (52.7)	0.00	227 (57.4)	318 (78)	0.00
Supply water (piped)	13 (2.9)	29 (6.7)		25 (6.3)	49 (12)	
Pond/ Filtering of pond water	69 (15.8)	129 (29.7)		115 (34)	36 (8.8)	
Others	36 (8.3)	47 (10.8)		10 (2.6)	13 (3.2)	
Toilet facilities, *n* (%)						
Sanitary with flush (water sealed)	118 (27.1)	133 (30.6)	0.09	119 (29.7)	117 (28.6)	0.53
Sanitary without flush (water sealed)	246 (56.6)	218 (55.1)		244 (61.7)	261 (63.8)	
Pucca/pit (not water sealed)	49 (11.3)	47 (10.8)		30 (7.6)	30 (7.3)	
Kaccha/hanging (fixed place)	21 (4.8)	37 (8.5)		4 (1.1)	1 (0.2)	

Decimal^a^: A decimal is a unit of area approximately equal to 1/100 acre (40.46 m^2^). 1decimal equals to 435.6 square feet.

### Operational efficiency and consumption of VGD rice

Table 4 demonstrates the operational efficiency, consumption, amount of rice the beneficiaries received every month and whether they consumed all their rations or not. A recall period of 2 months prior to data collection was used to capture this information. Most of the respondents in the FFR group received the fortified rice and the non-FFR group received the normal rice. Majority of the respondents received their entitled rice every month in the FFR group. In contrast to the non-FFR group, around half of the beneficiaries received rice every month during the 12-month period. All respondents of the FFR group received sealed packs compared to 25% in the non-FFR group. Around 20% of the respondents in the non-FFR group reported they did not receive the full 30 kg of rice per month compared to 8% in the FFR group. It is important to note that at endline, almost 80% of the households needed to buy extra rice from the local market in the last two months, with more than 75% of the households in both groups buying more than 30 kg of rice in last two months. The amount of rice was not sufficient to fulfil the requirements for all family members for the whole month which indicates the need to buy the extra normal rice.

**Table 4:** Operational efficiency and consumption of VGD rice.

Indicator	FFR	Non- FFR	*P-value*
	N = 397 n (%)	N = 409 n (%)	
**Type of rice received**			
Normal Rice (non-FFR)	11 (2.8)	403 (98.5)	0.00
Fortified rice (FFR)	380 (95.7)	6 (1.5)	
**Frequency of rice received in last 12 months**			
Once	6 (1.5)	49 (12.0)	0.00
Two times	1 (0.3)	79 (19.3)	
Three–six times	0 (0.0)	67 (16.4)	
Every month	390 (98.2)	214 (53.3)	
**Mode of packaging of VGD rice**			
In sealed pack	397 (100.0)	293 (71.6)	0.00
In sack brought from home	0 (0.0)	92 (22.5)	
Other pack	0 (0.0)	24 (5.9)	
**Any seal in the package mentioning the amount of rice (30 kg)**			
Yes	394 (99.2)	72 (24.6)	0.00
No	3(0.76)	221(75.4)	
**Average amount of rice usually received (kg) each month by women, *n* (%)**			
Less than 30 kg	33 (8.3)	79 (19.3)	0.00
Exactly 30 kg	364 (91.7)	330 (80.7)	
**Use of rice, *n* (%)**			
Consumed fully	386 (97.2)	397 (96.8)	0.64
Consumed partially	7 (1.7)	7 (1.7)	
Shared	2 (0.5)	1 (0.2)	
Sold	2 (0.5)	2 (0.5)	
Did not consume at all	0 (0.0)	2 (0.5)	
**Amount of VGD rice consumed by the household members in last two months (kg), *n* (%)**			
0–29 kg	1 (0.3)	3 (0.7)	0.00
30–60 kg	396 (99.7)	406 (99.2)	
**Purchasing of Extra rice in last two months, *n* (%)**			
Bought extra rice in last two months	315 (79.35)	332 (81.2)	0.51
Did not buy extra rice in last two months	82 (20.6)	77 (18.8)	
**Amount of extra rice bought in last two months (in kg), *n* (%)**			
0–29 kg	72 (22.9)	76 (22.9)	0.99
30+ kg	243 (77.1)	256 (77.1)	
**Number of times extra rice was bought in last two months, *n* (%)**			
1–12 times	307 (97.5)	324 (97.6)	0.91
12–24 times	8 (2.5)	8 (2.4)	

### Micronutrient and morbidity status

The anaemia and zinc status of the women who consumed either FFR or non-FFR over 12 months is shown in Table 5. In baseline, 39% of the FFR group and 34% of the non-FFR group had anaemia. At endline, 34% of the women in the FFR group and 41% in the non-FFR group had anaemia. The DID estimation showed that the reduction in anaemia after 12 months of FFR consumption was significant (*p* = 0.035). At the end of the study, the mean haemoglobin levels were 12.06 g/dL in the FFR group and 11.84 g/dL in the non-FFR group. The DID in mean haemoglobin level after 12 months of FFR consumption was also statistically significant (*p* = 0.002).

**Table 5:** Anaemia, zinc deficiency and morbidity at endline.

	Baseline			Endline			DID^b^	*P-*value
Characteristic	FFR	Non-FFR	Diff^a^	FFR	Non-FFR	Diff^a^		
Anaemic (< 12 g/dL, *n* (%)	162 (38.8)	151(34.0)	4.8	128 (34.0)	157 (40.7)	-6.7	-11.5	0.035
Mean haemoglobin, g/dL	12.15	12.28	-0.13	12.06	11.84	0.22	0.35	0.002
Zinc deficient (< 10.1 mmol/L), *n* (%)	173 (37.2)	186 (37.6)	-0.4	118 (31.2)	144 (37.2)	-6	-5.6	0.062
Mean serum zinc, mmol/L	10.8	10.6	0.2	10.8	10.7	0.1	-0.1	0.6
**Morbidity (last two weeks)**								
Diarrhoea^c^	56 (12.87)	65 (14.94)	-2.07	31 (7.6)	41 (10.3)	-2.7	0.63	0.07
Fever^d^	186 (42.76)	171 (39.31)	3.45	105 (26.5)	135 (33.3)	-6.8	10.3	0.023
Inflammation^e^ [CRP (>10.0 mg/l)]	16(4.1)	14 (3.9)		15(4.0)	16 (4.1)			

Diff^a^ = difference between FFR and non-FFR at baseline and endline

DID^b^ = difference in difference

Diarrhoea^c^ was defined as three or more abnormally loose or liquid stools without blood in the last 24 hours

Fever^d^ is when a human’s body temperature goes above the normal range of 36–37 °C (98–100 °F)

Inflammation^e^ = The adjustment for elevated CRP (>10.0 mg/l) was done by a mathematical correction

Around 31% of the VGD women in the FFR group and 37% of the non-FFR group were zinc-deficient at endline. The DID estimation showed the prevalence of zinc deficiency decreased by 6% in the FFR group at the endline survey, though the effect of consuming FFR for 12 months on the prevalence of zinc deficiency was not statistically significant. The DID estimation also indicated that FFR did not significantly increase serum zinc concentrations.

About 10% of the respondents in both groups reported they had suffered from diarrhoea in the two weeks before the baseline survey. Women in the FFR group experienced significantly less episodes of diarrhoea and fever during the two-weeks, pre-endline data collection, compared to women in the control group. The DID estimation showed that consumption of FFR for 12 months reduced both diarrhoeal and fever (*p* = 0.023) morbidity. The CRP level remained similar during both baseline and endline among both groups.

### Factors associated with anaemia and zinc deficiency

Backward elimination multiple logistic regression was used to identify factors associated with anaemia and zinc deficiency (Table 6). The factors associated with anaemia were consumption of non-FFR, animal protein intake (calculated by using the food frequency questionnaire for intake of animal protein), geographical region, respondent’s age and heavy menstrual flow. The non-FFR group had 1.33-fold higher odds of being anaemic compared to the FFR group. Older women had significantly greater odds of developing anaemia compared to women below 35 years of age. Women who suffered heavy menstrual bleeding also had higher odds of developing anaemia (aOR = 1.61; CI: 1.09–2.42, *p* = 0.017). Respondents in Gopalgonj district had a higher rate of anaemia than women in Gazipur district. Logistic regression indicated that women from the non-FFR group had a higher odds of zinc deficiency (aOR = 1.35, CI: 1.02–1.77, *p* = 0.035).

**Table 6:** Factors associated with anaemia and zinc status among respondents.

	Anaemia		Zinc deficiency	
Covariates	Adjusted OR^a^ (95% CI^d^)	*P-*value	Adjusted OR^a^ (95% CI^d^)	*P-*value
Group				
FFR^b^	Ref		Ref	
Non-FFR^c^	1.33 (1.01,1.72)	0.04	1.35 (1.02,1.77)	0.03
Animal protein intake				
> 6 days	Ref		Ref	
No intake	0.74(0.41,1.32)	0.30	0.79 (0.44,1.42)	0.43
1–3 days	0.71(0.51,1.00)	0.05	0.99 (0.71,1.39)	0.95
4–6 days	0.78(0.57,1.08)	0.14	0.97 (0.70,1.35)	0.87
Drinking water				
Tap water	Ref		Ref	
Tube well	0.74 (0.41,1.35)	0.33	1.76 (1.01, 3.07)	0.04
River/canal	0.97(0.65,1.46)	0.90	1.13 (0.76,1.69)	0.53
Geographical region				
Gazipur	Ref		Ref	
Bagherhat	0.97 (0.60,1.59)	0.92	0.93(0.57,1.51)	0.77
Gopalganj	1.67(1.15,2.41)	0.00	0.93 (0.64,1.36)	0.72
Khulna	0.94(0.59,1.51)	0.79	1.03 (0.65,1.63)	0.90
Satkhira	1.13 (0.70,1.84)	0.62	0.93 (0.57,1.51)	0.77
Respondent age (years)				
15–25	Ref		Ref	
25–35	1.15 (0.67, 1.97)	0.60	1.22 (0.72, 2.09)	0.45
35–45	1.72 (1.00, 2.97)	0.04	1.21 (0.71, 2.09)	0.48
> 45	2.18 (1.15, 4.12)	0.02	1.48 (0.78, 2.82)	0.23
Menstrual problems				
Normal flow	Ref		Ref	
Heavy menstrual period	1.61 (1.09,2.42)	0.017	1.04 (0.70, 1.55)	0.84
Wealth index				
Highest	Ref		Ref	
Fourth	0.92 (0.62, 1.36)	0.67	1.66 (1.06,2.60)	0.02
Middle	0.76 (0.51, 1.15)	0.19	1.23 (0.79,1.91)	0.36
Second	0.81 (0.54, 1.23)	0.33	1.45 (0.95,2.22)	0.08
First	0.90 (0.59, 1.38)	0.63	1.72 (1.14,2.59)	0.01

Adjusted OR^a^ = Adjusted odds ratio

FFR^b^ = fortified rice

Non-FFR^c^ = non- fortified rice

CI^d^ = confidence interval

## Discussion

The purpose of this study was to assess the potential contribution of fortified rice in reducing the prevalence of micronutrient deficiencies, namely anaemia and zinc deficiency, among the vulnerable women of reproductive age group participating in the VGD programme in Bangladesh. The results indicated that provision of fortified rice through the VGD programme significantly reduced anaemia, but zinc deficiency was not affected.

After 12 months of fortified rice consumption, fewer women in the FFR group were anaemic compared to those in non-FFR group (34% vs. 41%). Furthermore, anaemia status improved in the FFR group but deteriorated in the non-FFR group between baseline and endline. The DID estimation showed consumption of fortified rice for 12 months significantly reduced the prevalence of anaemia. A study in Mexico showed consumption of iron-fortified rice for 6 months increased haemoglobin in women [12] and a study in Cambodia showed iron-fortified rice improved iron status among children aged between 6–16 years compared to a control group consuming non-fortified rice [30]. In Costa Rica, fortification of staple foods improved iron status among women (15 to 45-years-old) and children (1 to 7-years-old). The same study indicated that even though iron was the most likely micronutrient responsible for the reduction in anaemia, fortification with other micronutrients such as folic acid, vitamin B12 and vitamin A also reduced anaemia [31].

The prevalence of anaemia in the FFR group is greater than the value reported in the 2011 National Micronutrient Survey. This difference may be due to assessment of a group more vulnerable than the average population, i.e. VGD beneficiaries, in this study. The amount of fortified rice provided by the VGD programme appeared to be inadequate, as the majority (80%) of VGD households needed to buy extra rice from local markets. Iron deficiency is the most common cause of anaemia and is thought to be responsible for approximately 50% of cases of anaemia in Bangladesh [31–40]. However, a recent study conducted by Merrill et al. and the national micronutrient survey suggest there are other causes of low haemoglobin [5,41]. A high iron level in ground water has indicated iron deficiency may not be the major cause of low haemoglobin levels in Bangladesh [5,41]. We did not measure plasma ferritin or soluble transferrin receptor levels in this study. Therefore, we cannot confirm that iron deficiency is the major cause of anaemia among VGD women. Still, this study suggests that multiple risk factors exist for anaemia among the VGD women, including consumption of unfortified rice, older age, heavy menstrual bleeding and geographical region.

The results of the present study showed that older women (35–45 years and above) have a significant higher risk of anaemia compared to younger women. A systematic analysis on global burden anaemia suggests the contrary, where the prevalence indicates highest among under-5 age group and gradually decreasing with age in females [42]. Anaemia in elderly individuals have varied aetiologies, including inadequate nutrient intake, anaemia due to chronic disease or some unexplained cause or idiopathic [43]. Nevertheless, the cause of anaemia in around 33% of cases could not be explained, and there is generally just a mild decline in the haemoglobin level [44]. Unexplained anaemia may be due to a blunted erythropoietic response in the context of iron inadequacy, higher circulating levels of proinflammatory cytokines (e.g. interleukin-6, which may reduce erythropoietin levels), diminished androgen levels, and reduced proliferative and regenerative capacity of bone marrow stem cells [44]. Heavy menstrual bleeding is a common cause of iron deficiency anaemia among the premenopausal women and can negatively affect a woman’s quality of life. Thirty percent of women consider their menstruation to be heavy [45]. Although there is a large discrepancy between women’s perception of their menstrual loss and accurate measurement of blood flow, we found women who reported heavy menstrual bleeding had higher odds of developing anaemia.

Zinc is one of the most important micronutrients and is crucial for human metabolism, playing multiple roles in the maintenance of genetic material, including transcription of DNA, translation of RNA and cellular division [46]. Zinc status was not assessed in Bangladesh until 2011, when a nationwide micronutrient survey reported more than half of non-pregnant non-lactating mothers being zinc-deficient. Zinc deficiency remained the same in the non-FFR area between baseline and endline. One third of the FFR group were zinc-deficient at endline, and zinc deficiency reduced by 6% in the FFR area between baseline and the endline survey, implying fortified rice consumption contributed to elevated serum zinc levels. Although the DID analysis shows this change is not statistically significant, this trend indicates FFR consumption had a positive impact on zinc status. The risk factors for zinc deficiency among VGD women support this statement: the non-FFR group has significantly higher odds of low serum zinc than the FFR group. A previous study suggested that fortification with zinc and other micronutrients had no significant effect on haematological indicators [47]. A study of pregnant women in Argentina showed that in case of zinc and iron, the appropriate foods must be fortified, based on consideration of the bioavailability and interactions of these nutrients with the food matrix. Indeed, the amount of zinc ingested during pregnancy in that study remained below recommended levels [48]. Previous studies of zinc tablet supplementation reported an increase in serum zinc [49], improved development outcomes and a reduction in diarrhoeal morbidity [50,51]. However, the findings on the efficacy of zinc-fortified foods are not conclusive [51], and other studies failed to show an improvement in serum zinc after regular consumption of zinc-fortified foods [52–54]. One study in Thailand showed a remarkable improvement in serum zinc in young children (randomly selected school boys and girls of grades 1–3 and 4–6, mean age of 110±20.7 months) with low serum zinc at benchmark after general utilization of a zinc-containing, micronutrient-braced flavour blend [55,56]. The present study measured zinc deficiency at the community level in a less controlled setting, and absorption of zinc was not measured. Moreover, as consumption of FFR by the study participants was often inadequate, it is difficult to compare our results with other studies where the optimum/recommended levels of consumption were achieved [57].

Consumption of FFR reduced diarrhoeal and fever-related illnesses, as the FFR group reported less morbidity than the non-FFR group. Similarly, another study also reported reduced diarrhoeal morbidity after zinc supplementation [31]. Elevated CRP was not observed among the FFR group as in this study, the infection (CRP >10mg/L) rate was only 5% at both baseline and endline in the FFR group. Therefore, it is reasonable to conclude that these reductions in morbidity were due to consumption of fortified rice.

The most important conclusion of this effectiveness study is that fortified rice appears to beneficially reduce the prevalence of anaemia among vulnerable women in Bangladesh. Therefore, provision of fortified rice instead of regular rice can be recommended for the VGD programme as the large rice-based social safety nets operated by the Government of Bangladesh can be the ideal entry point to address micronutrient deficiencies at scale in Bangladesh. In a potential next survey, there should be more time between the baseline and endline measurement to allow the effect of fortified rice on micronutrient status to be measured over a longer period. Furthermore, the high prevalence of anaemia in the control area indicates closer attention should be paid to promote effective behaviour change communication (BCC) to improve dietary diversity.

## Strengths and limitations of the study

The key strength of our study is the study design and large sample size. This effectiveness study followed a very robust methodology. This is the first study that has been attempted to identify that the food based social safety net programme like VGD which not only improves food security, extreme poverty, hunger and livelihood, but also helps to reduce micronutrient deficiencies in high-risk groups in Bangladesh, with a strong focus on women and children, through the consumption of fortified rice. The observed operational efficiency (in terms of quality, quantity, frequency) in FFR distribution programme will support the relevant stakeholders to improve the implementation process and compliance of the regular VGD operation. As a preselected listing of eligible VGD women was used for the sampling frame for this effectiveness study, there was limited scope to balance the socio demographic background for the FFR and non- FFR group during baseline and endline study. There is sufficient evidence that large inclusion bias and geographic mistargeting in the VGD programme may have an inefficient selection process [58]. If the inclusion of ineligible persons in the scheme were reduced, it could have increased the balanced allocation.

## Conclusion

This community-based intervention study indicates consumption of fortified rice significantly contributed to reducing the prevalence of anaemia to an extent among vulnerable women of reproductive age in Bangladesh. This fortified rice intervention could be incorporated with existing programmes that have scope for a food distribution component to improve the micronutrient status of the general population in Bangladesh.

## Supporting information

S1 Dataset PlosOne.dta. (DTA)
